# Industrial, CBD, and Wild Hemp: How Different Are Their Essential Oil Profile and Antimicrobial Activity?

**DOI:** 10.3390/molecules25204631

**Published:** 2020-10-12

**Authors:** Valtcho D. Zheljazkov, Vladimir Sikora, Ivayla Dincheva, Miroslava Kačániová, Tess Astatkie, Ivanka B. Semerdjieva, Dragana Latkovic

**Affiliations:** 1 Crop and Soil Science Department, 3050 SW Campus Way, Oregon State University, Corvallis, OR 97331, USA; 2 Institute of Field and Vegetable Crops, Alternative Crops and Organic Production Department, Maksima Gorkog 30, 21000 Novi Sad, Serbia; vladimir.sikora@ifvcns.ns.ac.rs; 3 Plant Genetic Research Group, Agrobioinstitute, Agricultural Academy, 8 “Dragan Tsankov” Blvd., 1164 Sofia, Bulgaria; ivadincheva@yahoo.com; 4 Department of Fruit Science, Viticulture and Enology, Faculty of Horticulture and Landscape Engineering, Tr. A. Hlinku 2, Slovak University of Agriculture in Nitra, 949 76 Nitra, Slovakia; kacaniova.miroslava@gmail.com; 5 Department of Bioenergetics and Food Analysis, Institution of Food Technology and Nutrition, University of Rzeszow, Cwiklinskiej 1, 35-601 Rzeszow, Poland; 6 Department of Engineering, Faculty of Agriculture, Dalhousie University, Truro, NS B2N 5E3, Canada; astatkie@dal.ca; 7 Department of Botany and Agrometeorology, Faculty of Agronomy, Agricultural University, 4000 Plovdiv, Bulgaria; v_semerdjieva@abv.bg; 8 Department of Field and Vegetable Crops, University of Novi Sad, 21000 Novi Sad, Serbia; dragana.latkovic@polj.uns.ac.rs

**Keywords:** *Cannabis sativa*, essential oil, cannabinoids, cannabidiol, δ9-tetrahydrocannabinol, dronabinol, monoterpenes, sesquiterpenes, wild hemp, hemp cultivars

## Abstract

Hemp (*Cannabis sativa* L.) is currently one of the most controversial and promising crops. This study compared nine wild hemp (*C. sativa* spp. *spontanea* V.) accessions with 13 registered cultivars, eight breeding lines, and one cannabidiol (CBD) hemp strain belonging to *C. sativa* L. The first three groups had similar main essential oil (EO) constituents, but in different concentrations; the CBD hemp had a different EO profile. The concentration of the four major constituents in the industrial hemp lines and wild hemp accessions varied as follows: β-caryophyllene 11–22% and 15.4–29.6%; α-humulene 4.4–7.6% and 5.3–11.9%; caryophyllene oxide 8.6–13.7% and 0.2–31.2%; and humulene epoxide 2, 2.3–5.6% and 1.2–9.5%, respectively. The concentration of CBD in the EO of wild hemp varied from 6.9 to 52.4% of the total oil while CBD in the EO of the registered cultivars varied from 7.1 to 25%; CBD in the EO of the breeding lines and in the CBD strain varied from 6.4 to 25% and 7.4 to 8.8%, respectively. The concentrations of δ9-tetrahydrocannabinol (THC) in the EO of the three groups of hemp were significantly different, with the highest concentration being 3.5%. The EO of wild hemp had greater antimicrobial activity compared with the EO of registered cultivars. This is the first report to show that significant amounts of CBD could be accumulated in the EO of wild and registered cultivars of hemp following hydro-distillation. The amount of CBD in the EO can be greater than that in the EO of the USA strain used for commercial production of CBD. Furthermore, this is among the first reports that show greater antimicrobial activity of the EO of wild hemp vs. the EO of registered cultivars. The results suggest that wild hemp may offer an excellent opportunity for future breeding and the selection of cultivars with a desirable composition of the EO and possibly CBD-rich EO production.

## 1. Introduction

Hemp (*Cannabis sativa* L.) is a new-old crop, one of the most controversial and promising crops due to its multiple utilizations, and contains a wide array of biologically active substances synthesized and accumulated in different plant parts [1]. Industrial hemp has been grown for grain and fiber for many decades in Europe, Asia, and North America. In addition, there is wild hemp (*C. sativa* spp. *spontanea* Vavilov, also known as spontaneous), which is native to both Central and Eastern Europe as well as parts of Asia, and is found as a weed in agricultural fields.

Hemp essential oil (EO) can be extracted using various extraction methods, the simplest and most natural using either steam or hydro-distillation, as is the case with many other EO crops. Hydro-distilled or steam distilled hemp EO are generally preferred by consumers and can be incorporated into a number of certified organic products; the market for organic food and non-food products reached US$55 billion in 2019 in the USA alone [2]. Currently, cannabidiol (CBD) production and markets in many countries are depressed due to the 2019 overproduction and the COVID-19 situation. For example, hemp production in the USA has increased rapidly and by mid-2019, there were around 500,000 licensed acres to grow hemp [3]. That acreage in 2019 would have generated an estimated US$11.3 billion income, or around 6% of the total value of all cash crops in the USA [3]. However, due to the depressed markets and expensive feminized seed (US$1/seed), there is now significant interest in hemp EO from industrial hemp cultivars and even from wild hemp. Indeed, a number of producers and processors in the USA are developing new products based on naturally extracted hemp EO or cannabinoids. Overall, research suggests that the hemp EO has medical significance and may also be utilized as an ingredient in commercial insect repellents and biopesticides [4,5]. Some cultivars such as Finola have been employed for commercial production of EO, as hemp EO has commanded high prices in recent years.

Hemp terpenes in the EO contribute to the aroma of various cannabis genotypes, and so far, around 140 different terpenes have been reported in this plant [1,6,7,8]. Current thinking is that terpenes have played a key role in the selection of medical/recreational and CBD type cannabis because their concentration is positively correlated to some of the cannabinoids [9].

The hemp EO profile depends on genotype, growth conditions, and extraction method (steam, hydro, CO_2_, or solvent extraction) [10]. Of the various groups of terpenes, monoterpenes such as limonene, β-myrcene, α-pinene, β-pinene, and linalool (containing 10 carbon units) comprise the major portion of the volatile oil fraction [10]. These EO constituents are widely found in the EO of other plant species such as spices, EO crops, and medicinal herbs; they are not specific to cannabis. Sesquiterpenes (with 15 carbons) such as β-caryophyllene, α-humulene, caryophyllene oxide, and β-phellandrene are also present in higher concentrations in hemp extracts [9,10]. Monoterpene composition can distinguish between monoecious and dioecious hemp cultivars [10]. Furthermore, certain terpenoids are highly correlated to the concentration of CBD and Δ9-tetrahydrocannabinolic acid (THCA); consequently, they have been proposed as a chemotaxonomic classification tool and to distinguish drug-type cannabis in Nevada [11]. Overall, hemp varieties (cultivars) with a higher concentration of monoterpenes have a more pleasant aroma compared with the varieties with higher amounts of sesquiterpenes. The monoterpenes pinene and limonene are the determinants of cannabis aroma in the immediate vicinity of the plant [12]. Hydro-distilled EO from industrial hemp cultivars may contain CBD [10]; the interaction of environment and genetics plays a role in the hemp EO profile.

Hemp EO (distilled from leaves, inflorescences, and thinner stems) has shown biological activity against several targets of pharmaceutical interests *S. aureus*, *H. pylori*, *Candida*, and *Malassezia* spp., enzymes, and cancer cell lines [13]. The EOs (collected from inflorescences after blooming) of cvs. Carmagnola, Fibranova, and Futura have shown significant antimicrobial activity against G^+^ and G^-^ bacteria and yeast, and the effect depended on the cultivar and seeding date [14].

This study addresses a knowledge gap and current industry interest towards hemp EO with different origins and profile. The hypothesis was that wild hemp would have a different EO content, composition, and antimicrobial activity compared with the EOs of registered industrial hemp cultivars, new hemp breeding lines, and a hemp strain (unregistered cultivar) that is currently used for the commercial production of CBD. The objective of this study was to compare nine wild hemp accessions (*C. sativa* spp. *spontanea*) sampled from agricultural fields in northeastern Serbia with 13 EU registered cultivars, eight breeding lines, and one CBD hemp strain (belonging to *C. sativa*) with respect to their EO profile and antimicrobial activity.

## 2. Results and Discussion

### 2.1. Essential Oil (EO) Content (Yield)

The EO yield of the wild hemp accessions varied from 0.085 to 0.262 mL/100 g for air-dried material and the yield of the breeding lines was 0.06 to 0.14, while the EO yield of the registered cultivars was 0.1 to 0.2 mL/100 g of dried material. However, the overall differences in oil yield between the three groups of hemp were not significantly different, with an overall mean of 0.129 mL/100 dried material (Table 1).

Overall, the EOs of the wild hemps and registered cultivars in this study were similar to those reported previously: 0.23 to 0.31% in fresh inflorescences [14], 0.29 to 0.19% depending on the collection time with higher EO yield from plants sampled earlier (in September than in October) [13], and 0.1% in stems and 0.15% in the leaves of wild hemp from Austria [15], respectively. However, the EO content of the USA hemp strain, utilized for CBD production in the USA and grown near the field trials was 1.15 to 1.2%.

### 2.2. Essential Oil (EO) Profile of the Three Groups of Hemp

For the wild hemps, two locations (Kovacica and Susara) were randomly selected among the nine locations (Slavka, Kovacica, Buro, Daleka zemlia, Susara, Saykaj, Perez, Titelski breg, and Paluka) to be used as two replications to represent wild hemp in the statistical analyses. As indicated in the Materials and Methods section, one-way ANOVA was completed to determine the significance of differences between the mean constituents obtained from nine hemp cultivars from Northeast Serbia. The constituents were: α-pinene, β-pinene, isocaryophyllene (γ-caryophyllene), β-caryophyllene, α-(E)-bergamotene, (Z)-β-farnesene, caryophyllene oxide, humulene epoxide 2, selina-6-en-4-ol, caryophylla-4(12),8(13)-dien-5α-ol, caryophylla-4(12),8(13)-dien-5β-ol, 14-hydroxy-(Z)-caryophyllene, β-bisabolol, α-bisabolol, CBD, and δ9-tetrahydrocannabinol.

Overall, the EO profile of the wild hemp was different from that of one of the registered cultivars and the new breeding lines (Supplementary Tables S1–S3). The EO constituents whose concentrations were significantly different are shown in bold in Table 1. The mean concentrations of the five constituents and CBD from the above list that did not have significant differences between the wild and the registered cultivars are shown in Table 1. The 10 constituents whose means were significantly different are shown in Table 2. The comparative concentrations of the EO constituents in this and previous reports cited here are summarized in Table 3.

#### 2.2.1. α-Pinene

The concentration range of α-pinene (bicyclic monoterpene) in the EO was from non-detected (n.d.) in three accessions of wild hemp to 2.5% in wild hemp Slavka; from n.d. to 8.4% in the registered cultivars; and 0.15 to 2.95% in the new breeding lines (Supplementary Tables S1–S3). However, when grouped together, the concentration of α-pinene in the EO was not statistically different between the three groups of hemp. The concentration of α-pinene in the EO of the USA hemp strain (grown in the vicinity of the registered cultivars in this study and extracted the same way for the same time duration) was 3.6 to 4.5% of the total EO. The monoterpenes pinene and limonene are the determinants of cannabis aroma in the immediate vicinity of the plant [12]. α-Pinene concentration in the EO in this study was comparable to previous reports from studies that used either steam or hydro-distillation [15,16,17,18], but was lower than that in other reports [14,17,19] (Table 3). The EO of wild hemp from Austria contained 20% of this compound in stems, but a much lower concentration in the leaves [15], while the EO of spontaneous (wild) hemp from Hungary contained 1.6, 2.9, and 0.7% of this compound in the leaves, male, and female flowers, respectively. These differences may be due to the environment (growing conditions including latitude and altitude), the plant parts analyzed, and/or the genetics (cultivar).

#### 2.2.2. γ-Caryophyllene (Bicyclic Sesquiterpene)

The concentration range of γ-caryophyllene in the EO was 0.2 to 1.23% in wild, 0.6 to 1.4% in the registered cultivars, and 0.57 to 1.6% in the EO of the breeding lines (Supplementary Tables S1–S3). γ-Caryophyllene concentration in the EO of the USA strain was 0.12%.

#### 2.2.3. β-Caryophyllene (Bicyclic Sesquiterpene)

The concentration of β-caryophyllene was 15 to 30% in the EO of wild hemp, 22 to 55% in the registered cultivars, and 11 to 22% in the EO of the breeding lines (Supplementary Tables S1–S3). Overall, statistically, the highest concentration of β-caryophyllene was found in the EO of cv. Spic and the lowest in the EOs of cvs. Simba and Dioica (Table 2). β-caryophyllene in the USA strain EO was 6.8 to 7.5%. β-Caryophyllene in the EO of this study was similar to those reported in previous studies [10,12,14,16,20,21] (Table 3). The data from this study and previous reports suggest that the concentration of β-caryophyllene could vary significantly depending on the cultivar. (E)-β–caryophyllene is known as the major EO constituent in hemp [21]. This is one of the *C. sativa* EO caryophyllane- and humulane-type sesquiterpenes that include sesquiterpenes (E)-β-caryophyllene, (Z)-β-caryophyllene, caryophyllene oxide, and the ring-opened isomer α-humulene (α-caryophyllene) [21]. This EO compound has been shown to function in vivo as a non-psychoactive CB2 receptor ligand in foodstuff [21].

#### 2.2.4. α-(E)-Bergamotene

The concentration of α-(E)-bergamotene (bicyclic sesquiterpene) ranged from 0.75 to 1.9% in the wild hemp, 0.36 to 4.4% in the registered cultivars, and 0.5 to 2.8% in the breeding lines (Supplementary Tables S1–S3). Overall, the concentration of α-(E)-bergamotene was statistically higher in cvs Bacalmas and Helena, and the lowest in cv. CS (Carmagnola Selezionata) (Table 2). The concentration of this compound in the USA strain EO was 0.95%. Overall, the concentration of this EO compound in wild hemp from this study was similar to that in the literature reports on wild hemp from Austria and Hungary [15,18].

#### 2.2.5. Caryophyllene Oxide

The concentration of caryophyllene oxide (bicyclic sesquiterpenoid) was 0.24 to 31% of the total EO in wild hemp, 3.9 to 6.8% in registered cultivars, and 8.9 to 17% in the EO of the breeding lines (Supplementary Tables S1–S3). The concentration of caryophyllene oxide was statistically higher in the EOs of cvs. Bacalmas, Helena, and Sequieni, and lowest in the EO of cv. Carmagnola (Table 2). The concentration of this compound in the USA strain EO was 1.3 to 1.4%. Caryophyllene oxide in the EO of wild hemp from Austria was 4.5% [15], while its concentration in the EO of spontaneous hemp from Hungary was 4.3, 4.5, and 2.3% in the leaves, male, and female flowers, respectively [18].

#### 2.2.6. Humulene Epoxide 2

The concentration of humulene epoxide 2 (bicyclic sesquiterpenoid) was 1.3 to 3.2% in wild hemp, 0.4 to 2.3% in registered hemp cultivars, and 2.3 to 5.6% in the EO of the breeding lines (Supplementary Tables S1–S3). Overall, the concentration of humulene epoxide 2 was statistically higher in the cvs. Bacalmas and Sequieni, and the lowest in cv. Spic (Table 2). The concentration of this compound in the USA hemp strain EO was 0.47%. The concentration of this compound in the EO of wild hemp from Austria was <2.5% [15], while in the EO of spontaneous hemp from Hungary, it was 1.2, 1.1, and 0.6% in the leaves, male, and female flowers, respectively [18].

#### 2.2.7. Selina-6-en-4-ol

Selina-6-en-4-ol (bicyclic sesquiterpenoid) was 0.23 to 1.6% in the EO of wild hemp, n.d. to 1.8% in the EO of the registered cultivars, and 1.1 to 2.8% in the EO of the breeding lines (Supplementary Tables S1–S3). Its concentration was statistically highest in the EO of cv. Dioica and lower in the EOs of cvs. Bacalmas and Spic (Table 2).

#### 2.2.8. Caryophylla-4(12),8(13)-dien-5β-ol

The concentration of caryophylla-4(12),8(13)-dien-5β-ol (bicyclic sesquiterpenoid) was 1.1 to 5.4% in the EO of wild hemp; from n.d. to 2.3% in the EO of the registered hemp cultivars; and 2.3 to 6.6% in the EO of the breeding lines (Supplementary Tables S1–S3). Its concentration was statistically the highest in the EO of the wild hemp and the lowest in the EO of cv. Spic (Table 2). The concentration of this compound in the USA strain EO was 0.85 to 0.88%, while its concentration in the EO of spontaneous hemp from Hungary was 2.3, 1.0, and 0.8% in the leaves, male, and female flowers, respectively [18].

#### 2.2.9. β-Bisabolol

The concentration of β-bisabolol (monocyclic sesquiterpenoid) was 0.9 to 4.5% in the EO of wild hemp; from n.d. to 1.8% in the EO of the registered cultivars; and 2.9 to 4.0% in the EOs of the breeding lines (Supplementary Tables S1–S3; Table 2). Overall, β-bisabolol was higher in the EOs of cvs. Bacalmas, Sequieni, and in the wild hemp, and the lowest in the EO of cv. Spic (Table 2). The concentration of this compound in the USA strain EO was 0.33%.

#### 2.2.10. α-Bisabolol

The concentration of α-bisabolol (monocyclic sesquiterpenoid) was 0.4 to 2.9% in the EO of wild hemp, n.d to 6.9% in the registered cultivars, and 0.5 to 3.5% in the EO of the breeding lines (Supplementary Tables S1–S3). α-Bisabolol was statistically the highest in the EO of cv. CS and lower in wild hemp and cvs. Bacalmas, Sequieni, and Spic (Table 2). α-Bisabolol in the USA strain EO was 3.0 to 4.4%. Epi-α-bisabolol was reported in the EO of spontaneous hemp from Hungary [18].

The major EO constituents of the USA hemp strain that was grown in close vicinity had a different chemical profile, with major constituents of myrcene (9.2 to 12%), β-caryophyllene (6.5 to 7.5%), limonene (3.8 to 4.2%), β-(E)-ocimene (5.3 to 5.6%), and α-bisabolol (3.9 to 4.4%). Some previous reports identified a different number of EO constituents in hemp EO (e.g., 55 EO constituents, with myrcene, α-pinene, and β-pinene as the main monoterpenes, and β-caryophyllene as the main sesquiterpene) [14]; 84 EO constituents were identified in wild (spontaneous) hemp by Nagy et al. [18]. The latter authors named these hemp plants spontaneous forms, with the main EO constituents of the leaves, male and female flowers being E-caryophyllene (28.3%), α-humulene (8.9 to 9.3%), β-selinene (4.3 to 7.3%), and α-selinene (2.9 to 5.1%) [18]. Apparently, the spontaneous hemp plants from Hungary had a different EO chemical profile compared with the EO of the wild hemp of this study that was collected in the northeastern part of Serbia. The hydro-distilled leaf EO of wild hemp in Austria contained mainly β-caryophyllene (26.2%), α-humulene (13.1%), β-selinene (5.0%), caryophyllenen oxide (4.5%), and α-selinene (4.4%) [15]. In the same study, stem EO contained α-pinene (20.2%), β-caryophyllene (8.3%), β-pinene (7.0%), and myrcene (6.1%) [15]. Apparently, the EO of wild hemp in Austria had a similar composition to some, but not to other wild hemp EOs, in this study.

Previous research has suggested that metabolic fingerprinting can be used for chemotaxonomic purposes in *C. sativa* [9]. However, this and earlier reports on wild hemp [15,18] have demonstrated that the EO profile of wild hemp can vary significantly. Therefore, genetic analyses may be needed to ascertain if the spontaneous or wild hemps in Europe originated from some of the old industrial hemp cultivars that have been grown in Europe over the last few centuries or from medical cannabis, or are products of spontaneous crossings between the two groups.

### 2.3. Cannabinoids in the EO of the Three Hemp Groups: Cannabidiol (CBD) and δ9-Tetrahydrocannabinol (THC) Content

#### 2.3.1. Cannabidiol (CBD, Cannabinoid)

In this study, the concentration of CBD in the EO of wild hemp varied from 6.9 to 52.4% of the total oil, the CBD in the EO of the registered cultivars was from 7.1 to 25.4%, while the CBD concentration in the oil of the breeding lines was from 6.4 to 25.4% (Supplementary Tables S1–S3). However, when we grouped them together, because of the high variation, there were no significant differences between the wild accessions, registered cultivars, and the breeding lines with overall means of 12.4% (Table 1). The concentration of CBD in the EO of the USA strain (which is commercially grown for CBD production) varied between 7.5 and 7.8% of the total EO, which is an interesting result. The CBD concentrations in the EO of wild hemp Slavka, Kovacica, Susara, Perez and Titelski breg were 14.9, 22.6, 11.3, 17.5, and 15.7%, respectively, while the CBD concentrations in the EO of Buro and Saykaj were 52.4 and 33.7%, respectively. Therefore, this and previous studies [15,18] support the notion that wild hemps can be used as a source for the commercial production of CBD-enriched EO. This is the first report on such a high concentration of CBD in hydro-distilled hemp EO.

#### 2.3.2. δ9-Tetrahydrocannabinol (THC, Cannabinoid) (Dronabinol)

Overall, the concentration of THC in the EO was significantly different between the three groups of hemp (Table 3). The THC concentration was significantly higher in the EO of wild hemp accessions, with an average of 2.4% of the total oil, and a range between n.d. (in Daleka Zemlia) and 3.4% (in Kovacica) (Supplementary Tables S1–S3). Interestingly, it was much higher than the THC (0.16%) in the EO of the USA hemp strain, which was actually developed from marijuana type hemp. The THC concentration in the EO of most of the registered cultivars varied from n.d. (e.g., in cvs. Spic and Bacalmas) to over 1.2% (in the EO of cv. Chameleon) (Table 2). The THC concentration in the EO of the breeding lines was generally low, n.d., or below 0.4% with the exception of line SK8, where it reached 3.6% of the total oil. Previous research has shown that hydro-distilled EO from industrial hemp varieties contained cannabidiol (CBD) [10].

### 2.4. Chemical Groups

Overall, the content of monoterpenes fluctuated from n.d. to 8.0% in the EO of wild hemp, 0.3 to 13% in the EO of the registered cultivars, and 0.2 to 10% of the EO of the breeding lines (Supplementary Tables S1–S3). Monoterpene concentration in the EO of the USA hemp strain was 33 to 34% of the oil.

Sesquiterpenes were the largest group of chemical constituents. The content of sesquiterpenes was 41 to 79% of the EO of wild hemp, 65 to 89% of the EO of the registered cultivars, and 70 to 89% of the EO of the breeding lines (Supplementary Tables S1–S3). Sesquiterpenes constituted 54 to 55% of the EO in the USA hemp strain.

Cannabinoids also comprised the second largest group of chemical constituents in the wild hemp. The concentration of cannabinoids was 7 to 56% of the EO of wild hemp, 4.6 to 28% of the EO of the registered cultivars, and 6.4 to 26% of the EO of breeding lines (Supplementary Tables S1–S3). Cannabinoid concentration in the EO or USA hemp strain were 8.2 to 8.5%, surprisingly low. Most of the strains for thee commercial production of CBD were selected from marijuana (drug-type hemp with 4 to 12% of Δ^9^-THC %), however, they were selected to have <0.3% total THC in dried biomass in order to be compliant with the current rules and regulations that are evolving [25].

In a study of spontaneous hemp, Nagy et al. [18] reported that the EO was majorly composed of sesquiterpene hydrocarbons (57.1 to 62.8%), followed by cannabinoids (11.0 to 29.3%) and oxygenated sesquiterpenes (7.8 to 14.8%). Overall, the results from this study suggest that wild/spontaneous hemp in Europe is chemotaxonomically related to the industrial hemp varieties (cultivars) grown in Europe and deviate from the chemical profile of the USA hemp strain that was developed from marijuana-type cannabis for the commercial production of CBD. The USA hemp strain used in this study was started with feminized seed, which guarantees the production of female only plants, which may be one of the reasons for its much higher EO content.

The pharmacological power of hemp is based on the content of Δ9-tetrahydrocannabinolic acid (THCA) and cannabidiolic acid (CBDA) [26]. Other major cannabinoids include cannabinolic acid (CBNA), cannabigerolic acid (CBGA), cannabichromenic acid (CBCA), and cannabinodiolic acid (CBNDA) [1,27]. Current hemp EO and cannabionoid production systems in the USA have been scaled up from marijuana production. Like with marijuana production, hemp ‘Christmas tree’ production systems rely on feminized seed, because female plants with non-fertilized flowers (flower bracts) accumulate significantly higher concentrations of secondary metabolites such as cannabinoids and terpenes compared with fertilized flowers [28]. Currently, hemp chemical production is based on non-registered hemp ‘strains’ that were originally developed by marijuana breeders, which must meet government regulations for hemp with less than 0.3% Δ9-tetrahydrocannabinolic acid (THCA) in the dried biomass. Due to the rapidly growing market for non-psychoactive cannabinoids, mainly cannabidiol (CBD), most of production has been focused on cannabidiolic acid (CBDA). Newer hemp strains bred for these characteristics are more likely to be compliant, and breeding companies are working on the registration of a number of hemp strains with high CBD content as commercial cultivars. The results from this study demonstrate that wild hemp may be a good source for further selection and breeding of cultivars with high concentration of CBD and low concentrations of THC.

### 2.5. Antimicrobial Activity of the Hemp Essential Oils (EO)

We used antibiotics as a positive control: cefoxitin for Gram-negative (G^-^) bacteria and gentamicin for Gram-positive (G^+^) bacteria, and fluconazole for yeast. From G^-^ we used SE, *Salmonella enterica* subsp. *enterica*; PA, *Pseudomonas aeruginosa*; and YE, *Yersinia enterocolitica*. From G^+^, we used SA, *Staphylococcus* subsp. *aureus*; EF, *Enterococcus faecalis*; and SP, *Streptococcus pneumoniae*. From yeast, CA, *Candida albicans*; CK, *Candida krusei*; and CT, *Candida tropicalis* were used. The method has been described previously [29].

The EOs of different hemp cultivars, accessions, and strains had differential antimicrobial activity that can be explained with differences in the EO profile. Some of the EOs had similar (although lower) activity to the antibiotics gentamycin, cetofoxin, and fluconazole.

**Figure 1:**
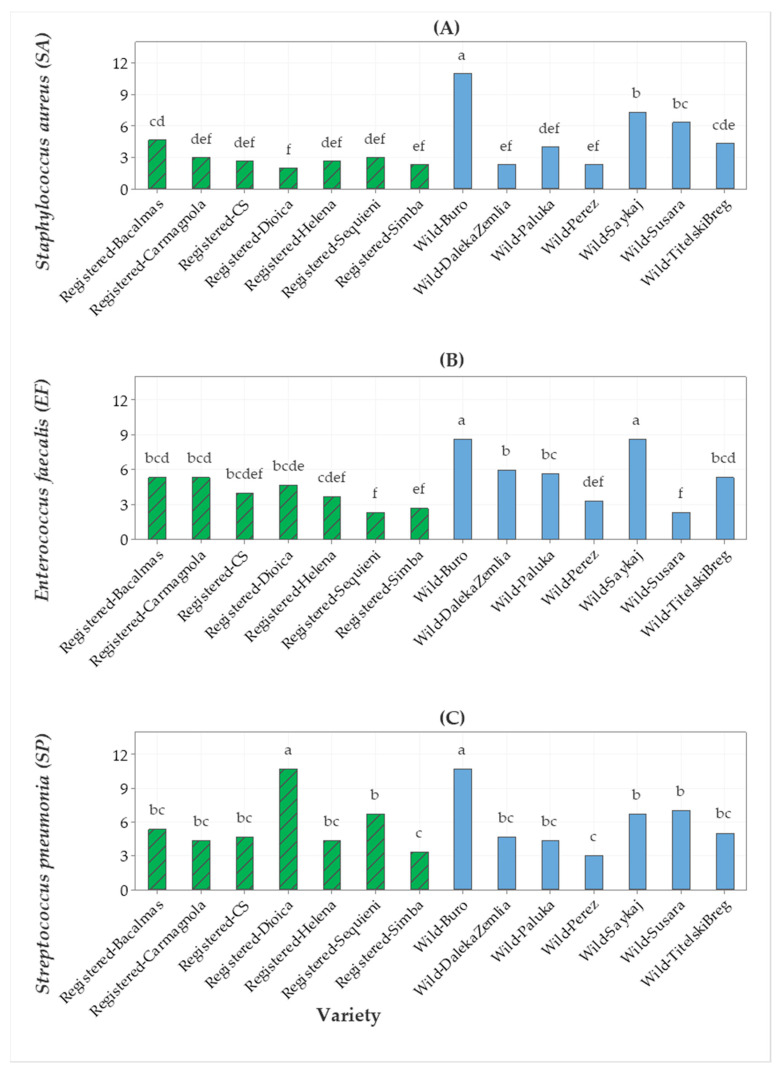
Bar graph of (**A**) *Staphylococcus* subsp. *aureus* (SA), (**B**) *Enterococcus faecalis* (EF), and (**C**) *Streptococcus pneumoniae* (SP) (gentamycin) antimicrobial activities (inhibition zone in mm) from seven registered cultivars and seven wild accessions. The means represented by the bars sharing the same letter are not significantly different.

The antibiotic gentamycin was used as a positive control in the data presented in Figure 1. The EO of wild hemp Buro was the most potent against *Staphylococcus* subsp. *aureus* (SA), followed by the EO of wild hemp Saykaj (Figure 1A). Similarly, the EOs of wild hemps Buro and Saykaj showed the highest antimicrobial activity against *Enterococcus faecalis* (EF). The EOs of wild hemp Buro and cv. Dioica were the most potent against *Streptococcus pneumoniae* (SP) (Figure 1B). The EOs of wild hemp Buro and the registered cv. Dioica had higher antimicrobial activity against *Streptococcus pneumoniae* compared with that of other EOs (Figure 1C).

**Figure 2:**
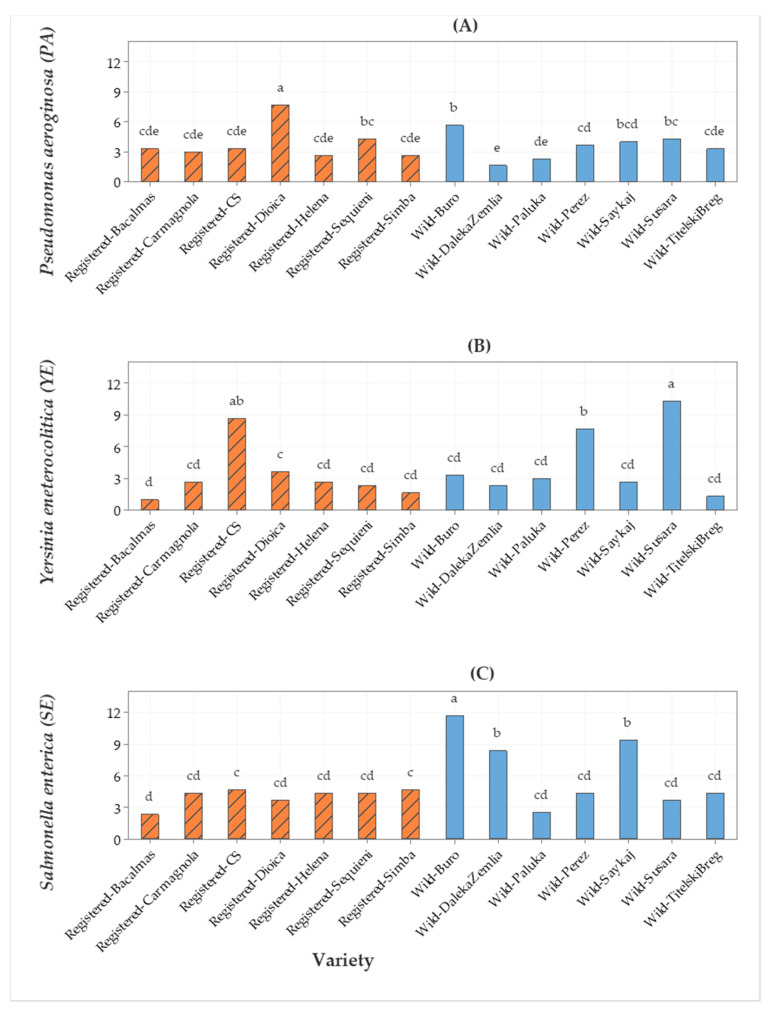
Bar graph of (**A**) *Pseudomonas aeruginosa* (PA), (**B**) *Yersinia* *enterocolitica* (YE), and (**C**) *Salmonella enterica* subsp. *enterica* (SE) (cefoxitin) antimicrobial activities (inhibition zone in mm) from seven registered cultivars and seven wild accessions. The means represented by the bars sharing the same letter are not significantly different.

Furthermore, the EOs of wild hemp Buro and the registered cv. Dioica had higher antimicrobial activity against *Pseudomonas aeruginosa* compared with that of the EOs of the other hemps (Figure 2A). Cetofoxin (antibiotic) was used as a positive control for the data in Figure 2. The EO of wild hemp Susara had the highest activity against the G^−^ *Yersinia enterocolitica* (Figure 2B). The EO of wild hemp Buro had the highest activity against the G^−^ *Salmonella enterica* subsp. *enterica*, lower than the EOs of wild hemps Daleka Zemlia and Saykaj, and the lowest in the EO of other hemps (Figure 2C).

**Figure 3:**
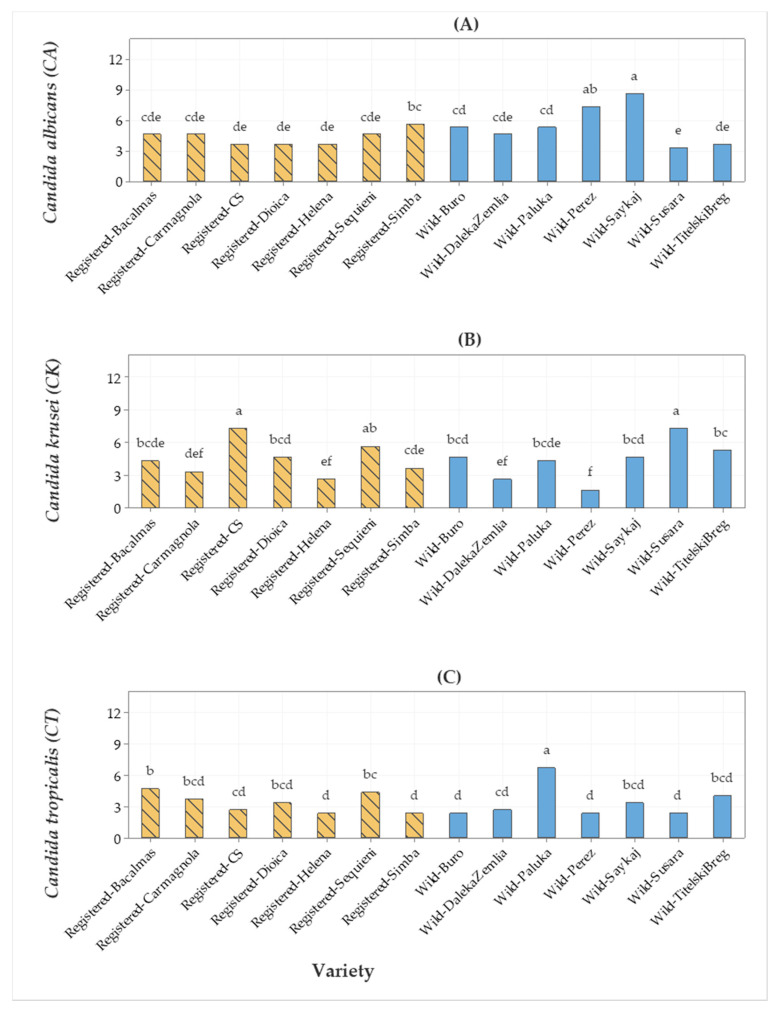
Bar graph of (**A**) *Candida albicans* (CA), (**B**) *Candida krusei* (CK), and (**C**) *Candida tropicalis* (CT) (fluconazole) antimicrobial activities (inhibition zone in mm) from seven registered cultivars and seven wild hemp accessions. The means represented by the bars sharing the same letter are not significantly different.

The antibiotic fluconazole was the positive control for the data presented in Figure 3. The EO of wild hemp Saykaj had the highest bioactivity against *Candida albicans* (CA), the bioactivity of EO of wild hemp Perez was not significantly different, while the bioactivity of the EOs of the other hemp EOs was lower than that of Saykaj (Figure 3A). The EO of wild hemp Susara and the EO of registered cv. CS had the highest bioactivity against *Candida krusei* (CK), while the bioactivity of the EO of Sequieni was not different from the above (Figure 3B). The EO of wild hemp Paluka had the highest bioactivity against *Candida tropicalis* (CT) (Figure 3C).

Previously, hemp EO has shown biological activity against several targets of pharmaceutical interest: *S. aureus*, *H. pylori*, Candida and Malassezia spp., enzymes, and cancer cell lines [13]. In another study, the EOs (collected from inflorescences after blooming) of cvs. Carmagnola, Fibranova, and Futura showed significant antimicrobial activity against Gram (+) and Gram (−) bacteria and yeast, and the effect depended on the cultivar and seeding date [14].

Overall, the findings in this study are consistent with the ones in a recent report [29]. This study provides new information on the antimicrobial activity of the EOs of the registered and wild hemps. Good antimicrobial activity against Enterococcus, Listeria, and Staphylococcus growth were found, which were compared to the conventional antibiotics that were used [30,31]. Novak et al. [31] found that the EO of five different cultivars of hemp had modest antibacterial activity. The Gram-positive bacterial strains tested demonstrated high sensitivity toward cannabidiol, with slightly lower effects by cannabidiolic acid [32]. Due to significant antimicrobial potency of CBD against MRSA, the synergy with conventional antibiotics was tested. However, CBD was not able to revert the resistance pattern or demonstrate synergy with any of the conventional antibiotics tested [33,34].

## 3. Materials and Methods

### 3.1. Plant Material

Certified industrial hemp (*Cannabis sativa* L.) seeds were obtained from the Institute for Field and Vegetable Crops in Novi Sad, Serbia. Field experiments were set up at the Alternative Crops and Organic Production Department in Backi Petrovac, Serbia (N502138 E395689) using 13 different cultivars of industrial hemp (Table 4), some of them included in the European List of Approved hemp varieties (cultivars) [35]. The cultivars used in this study included CS (Carmagnola Selezionata), Spic, Dioica, Helena, Carmagnola, Squieni, Bacalmas, Simba, Silesia, Chameleon, Fibrol, Futura, and Lovrin (Table 4). The results from the first eight varieties were used in the statistical analyses and in the tables. In addition, new hemp breeding lines (named SK1 to SK8) were seeded adjacent to the above experiment and subjected to the same growth conditions.

Although the botanical classification of hemp is controversial [36], wild populations belong to uncultivated narrow leaf *Cannabis sativa* ssp. *spontanea* Vavilov [37], which is considered native to Central and Eastern Europe and parts of Asia. Wild hemp samples in this study were collected from the edges of agricultural fields in the same region that have been used to grow other crops, but not hemp. These were agricultural fields where no hemp has been grown for the last 30 years. However, around 35–40 years ago, at some of the collection sites, there were hemp processing factories or hemp seed storage facilities. Generally, wild hemp is phenotypically different from plants of either old or new commercial hemp cultivars; wild hemp also has a shorter stature, with fewer branches than plants from registered cultivars. The locations of the wild hemp samples were named after the names of the nearby villages: Buro, Daleka Zemlia, Paluka, Perez, Saykaj, Susara, Slavka, Kovacica, and Titelski Breg (Table 5). The wild hemp samples were dried under the same conditions, and the EO was extracted via the same method and during the same time as the hydro-distillations of other hemp samples.

### 3.2. Performing Experiments

Hemp was grown as a rainfed crop without irrigation, as is traditional for the region, and the production technology, which is typical production system for commercial production of hemp in Middle and Southern Europe, was applied. Hemp was seeded with a corn planter on 27 March, 2019 with 50 cm spacing between rows and at a seeding rate of 30 kg/ha. The soil type was alluvial chernozem with pH 7.2., previous crop was millet. The soil preparation prior to seeding included deep plowing on 12 November, 2018 and fine seedbed preparation on 25 March, 2019. The experimental design was completely randomized with three replicates, with the size of the experimental plots being 2 m × 5 m. Fertilizer (NPK 16:16:16) was applied as a broadcast treatment at 300 kg/ha before deep plowing. An additional 50 kg/ha of nitrogen was applied in the spring before sowing. Weed control was conducted using burnout with glyphosate prior to seeding. Mechanized weed control was performed twice during the first four weeks of vegetation stage though the use of sweep-type cultivators. Hemp closes the canopy at 5-6 weeks after emergence and hence, suppresses weeds very well after that. The trial of the breeding lines was at the same field, approximately 60 m from the main trial, and was subjected to the same agricultural conditions. Plants in both trials were cultivated the same way including seeding time, seeding depth, fertilization, and weed control.

Fresh biomass samples (around 1 kg fresh, in three reps) were obtained on 27 June, 2019 at the beginning of flowering of the male plants. Hemp tissue samples were generated by cutting the top 1.5 feet (46 cm) from the top of female plants (male plants were not included in the samples). Fresh weight was measured, then the samples were hung in a shady area (tobacco dryer) until constant air-dried weight and then extracted.

### 3.3. Essential Oil (EO) Extraction

The EO extraction was conducted via hydro-distillation of hemp air-dried material using 4-L hydro-distillation Clevenger-type units as described previously for another plant material [38]. The sample size was up to 50 g dry weight (DW) biomass in 1.2 L water. All distillations were performed in two replicates, which was sufficient for statistical analyses. Beginning of the distillation was noted when the first drop of EO was deposited in the collection part of the Clevenger apparatus. All samples were distilled non-stop for 180 min. At the end of the distillation, the heat source was removed, the EOs (along with some water) were collected in glass vials, and placed in a freezer. After all distillations were complete, the EO was separated from water, measured on an analytical scale, and kept in a freezer until the gas chromatography (GC) analyses could be performed.

### 3.4. GC-FID (Gas Chromatography-Flame Ionization Detector) and GC-MS (Gas Chromatography–Mass Spectrometry) Analyses

Hemp volatile compounds were analyzed by GC-FID and GC-MS as described previously [38], with the following modifications: the column temperature was initially set at 40 °C, then increased to 300 °C at a rate of 5 °C/min, which was held for 10 min. The flow rate of the carrier gas (He) was maintained at 1.0 mL/min. The injection volume was 1.0 µL at a split ratio of 30:1. The temperatures of the ionization source, the transfer line, and the injector were 230 °C, 280 °C, and 250 °C, respectively. The MSD was operated in full scan mode. All mass spectra were acquired in electron impact (EI) mode with 70 eV in the *m*/*z* range of 40–400. The injector and detector temperature (FID) was set at 220 °C and 280 °C, respectively.

All constituents present in the EO samples were identified by comparing their linear retention indices (LRI) and MS fragmentation patterns with those from the National Institute of Standards and Technology (NIST′08) and Adams mass spectra library. The estimated LRI were determined using a mixture of a homologous series of aliphatic hydrocarbons from C_8_ to C_40_ under the same conditions described above.

### 3.5. Antimicrobial Assay

#### 3.5.1. Bacteria and Yeasts Culture

The microorganisms used for antimicrobial activity testing included Gram-negative bacteria *Pseudomonas aeroginosa* (CCM 1959), *Salmonella enterica* subsp. *enterica* (CCM 3807), *Yersinia enterocolitica* (CCM 5671); Gram-positive bacteria *Enterococcus faecalis* (CCM 4224), *Staphylococcus aureus* subs. *aureus* (CCM 4223), *Streptococcus pneumoniae* (CCM 4501); and yeasts *Candida albicans* (CCM 8186), *C. krusei* (CCM 8271), and *C. tropicalis* (CCM 8223) (Czech Collection of Microorganisms, Brno, Czech Republic). The bacteria cultures were incubated in Mueller Hinton broth (MHB, Oxoid, Basingstoke, UK) at 37 °C, but yeast cultures were in Sabouraud broth (SB, Oxoid, Basingstoke, UK) at 25 °C overnight.

#### 3.5.2. Disc Diffusion Method

For the agar disc diffusion method, a 100 µL of 10^6^ CFU/mL bacterial suspension after incubation was spread on the Mueller Hinton Agar (MHA, Oxoid, Basingstoke, UK). Filter paper discs (6 mm in diameter) were infused with 15 µl of the EO, tested, and placed on the inoculated MHA. MHA was kept at 4 °C for 2 h and then at 37 °C for 24 h. For yeasts, 100 µL of the yeast suspension was spread on Sabouraud agar (SA, Oxoid, Basingstoke, UK) and agars were cultivated at 25 °C for 24 h. After the incubation period, the diameter of inhibition zones was measured (mm). Growth inhibition was compared with the standard drugs. The standard drugs cefoxitin for G^−^ bacteria, gentamycin for G^+^ bacteria and fluconazole for yeasts were used as positive controls. Tests were performed in three separate experiments, and the means were calculated.

### 3.6. Statistical Analyses

One-way analysis of variance (ANOVA) with two replications was completed to determine the significance of differences among the mean oil content and constituents obtained from nine cultivars. The constituents were: α-pinene, β-pinene, isocaryophyllene (γ-caryophyllene), β-caryophyllene, α-(E)-bergamotene, (Z)-β-farnesene, caryophyllene oxide, humulene epoxide 2, selina-6-en-4-ol, caryophylla-4(12),8(13)-dien-5α-ol, caryophylla-4(12),8(13)-dien-5β-ol, 14-hydroxy-(Z)-caryophyllene, β-bisabolol, α-bisabolol, CBD, and δ9-tetrahydrocannabinol (Dronabinol). This completely randomized design has two replications.

The differences among the mean antimicrobial activities (SA, EF, SP, PA, YE, SE, CA, CK, and CT) obtained from the seven registered cultivars (Bacalmas, Carmagnola, CS, Dioica, Helena, Sequieni, and Simba) and seven wild accessions (Buro, Daleka zemlia, Paluka, Perez, Saykaj, Susara, and Titelski breg) were also compared using one-way ANOVA with three replications.

The analyses were completed using the mixed procedure of SAS [39]. Since the effect of cultivar was significant (*p*-value < 0.05) or marginally significant (0.05 ≤ *p*-value < 0.1) on most of the constituents, further multiple means comparison was completed using the lsmeans statement of Proc Mixed (equivalent to Fisher’s least significant difference [LSD]) at 5% level of significance and letter groupings were generated. The overall mean was calculated for the constituents where cultivar effect was not significant. For the antimicrobial activities, cultivar effect was highly significant (*p*-value < 0.01) on all nine activities, and considering the large number (14) of cultivars and accessions compared, multiple means comparison was done using Tukey’s multiple range test, which controls the Type II experiment-wise error rate.

For each response variable, the validity of model assumptions was verified by examining the residuals as described in Montgomery [40].

## 4. Conclusions

The results from this study demonstrated that the essential oil (EO) of wild hemp from Serbia is different in its chemical profile and bioactivity from the EO of registered industrial hemp cultivars, the breeding lines, and the hemp strain grown for CBD production in North America. Wild hemp EOs were also somewhat different from the EO profile of wild and spontaneous hemps collected in Austria and Hungary, as reported in the literature. However, although having been named differently, the wild hemp in this study, the wild hemp collected in Austria, and the spontaneous hemp from Hungary might have common origins; the collection sites were approximately in the same region (with a dimeter of approximately 500 km) although collected in three different countries. These wild/spontaneous hemps have been exposed to significant environmental and agricultural (pesticide) pressure over the last few decades. These populations may all belong to *Cannabis sativa* var. *spontanea* Vavilov, (a synonym of *Cannabis sativa* L.), considered native to Central and Eastern Europe and parts of Asia. However, the taxonomy of hemp is still debatable and there is no consensus among taxonomists as to whether it is a single species with several subspecies or multiple species.

The concentration of the four major constituents in the industrial hemp lines and wild hemp varied as follows: β-caryophyllene 11 to 22% and 15.4 to 29.6%; α-humulene (α-caryophyllene) 4.4 to 7.6% and 5.3 to 11.9%; caryophyllene oxide, 8.6 to 13.7% and 0.2 to 31.2%; humulene epoxide 2, 2.3 to 5.6% and 1.2 to 9.5%, respectively. The major EO constituents in the USA hemp strain that was grown in the vicinity of the field trials had different chemical profiles, with the major constituents myrcene (9.2 to 12%), β-caryophyllene (6.5 to 7.5%), limonene (3.8 to 4.2%), β-(E)-ocimene (5.3 to 5.6%), and α-bisabolol (3.9 to 4.4%).

Overall, the EO of wild hemp has shown greater antimicrobial activity against Staphylococcus susp. aureus, Enterococcus faecalis, and G−Yersinia enterocolitica, Salmonella enterica subsp. enterica, and yeasts Candida albicans and Candida tropicalis compared with the EO of registered cultivars.

This is the first report to show that a significant amount of CBD can be accumulated in the EO of wild and registered cultivars of hemp following hydro-distillation. One of the wild hemp EOs showed a very high concentration of CBD.

The EO of the wild hemp had a significantly higher concentration of THC relative to the one from the registered EU cultivars of industrial hemp breeding lines. In addition, it was interesting to see that wild hemp had a higher concentration of CBD in the EO relative to the EO from a strain grown for commercial production of CBD in the USA Therefore, wild hemp collected in Serbia could be used for the development of varieties (registered cultivars) with specific desirable chemical composition, and may also provide excellent material for the selection and breeding of hemp cultivars with high CBD for the commercial production of CBD and other high-value chemicals.

## Figures and Tables

**Table 1:** Analysis of Variance (ANOVA) *p*-values that show the significance of the effect of cultivar on 16 constituents, square root of mean squares error (Root MSE) that represents the common standard deviation, and the overall mean oil content (*v*/*w*, volume oil per dry weight) and concentration (%) of the five constituents with no significant difference between the cultivars and CBD. The *p*-values that show significant (*p* < 0.05) and marginally significant (0.05 ≤ *p* < 0.1) effect and need multiple means comparison are shown in bold.

Constituent	ANOVA *p*-Value	Root MSE	Overall Mean
Oil content	0.362	0.040	0.129
α-Pinene	0.439	2.083	2.148
β-Pinene	0.380	0.857	0.923
Isocaryophyllene (γ-Caryophyllene)	**0.072**	0.236	
β-Caryophyllene	**0.007**	2.768	
α-(E)-Bergamotene	**0.001**	0.431	
(Z)-β-Farnesene	0.141	0.878	1.669
Caryophyllene oxide	**0.052**	0.661	
Humulene epoxide 2	**0.001**	0.503	
Selina-6-en-4-ol	**0.064**	0.380	
Caryophylla-4(12),8(13)-dien-5α-ol	0.101	0.397	1.412
Caryophylla-4(12),8(13)-dien-5β-ol	**0.002**	0.303	
14-hydroxy-(Z)-Caryophyllene	0.212	0.263	0.349
β-Bisabolol	**0.001**	0.072	
α-Bisabolol	**0.003**	1.023	
CBD	0.487	5.574	12.39
δ9-Tetrahydrocannabinol (Dronabinol)	**0.001**	0.237	

**Table 2:** Mean concentration (%) of isocaryophyllene (γ-caryophyllene) [1], β-caryophyllene [2], α-(E)-bergamotene [3], caryophyllene oxide [4], humulene epoxide 2 [5], selina-6-en-4-ol [6], caryophylla-4(12),8(13)-dien-5β-ol [7], β-bisabolol [8], α-bisabolol [9], and δ9-tetrahydrocannabinol (dronabinol) [10] obtained from the 9 cultivars.

	Cultivar								
	Bacalmas	Carmagnola	CS ^1^	Dioica	Wild	Helena	Sequieni	Simba	ŠPIC
[1]	1.1 a	1.0 a	0.96 a	1.1 a	0.87 a	0.98 a	1.4 a	0.93 a	0.32 b
[2]	27 cd	27 cd	33 bc	26 d	28 bcd	28 bcd	33 b	25 d	40 a
[3]	3.1 a	2.1 bc	0.37 d	1.5 c	1.1 cd	3.8 a	1.2 cd	1.7 c	2.9 ab
[4]	6.6 a	4.2 c	5.4 abc	6.2 ab	5.6 abc	6.3 a	6.6 a	4.7 bc	6.0 ab
[5]	2.0 a	1.4 c	1.4 c	1.5 bc	1.7 ab	1.8 ab	1.9 a	1.6 bc	0.96 d
[6]	0.4 c	1.3 ab	0.56 bc	1.6 a	0.39 c	0.89 abc	0.49 bc	0.66 bc	0.19 c
[7]	1.8 bc	1.1 de	1.14 cde	1.5 cd	2.6 a	1.4 cd	2.3 ab	1.3 cd	0.55 e
[8]	1.7 a	1.1 cd	1.1 d	1.3 bc	1.8 a	1.38 b	1.7 a	1.1 cd	0.67 e
[9]	2.7 bcd	4.1 bc	6.7 a	2.1 bcd	1.6 d	1.75 cd	0.50 d	4.1 b	0.46 d
[10]	0.001 d	0.93 b	0.17 d	0.29 cd	2.4 a	0.14 d	0.42 bcd	0.76 bc	0.001 d

Within each row, means sharing the same letter are not significantly different. ^1^ CS—cv. Carmagnola Selezionata.

**Table 3:** Comparative concentrations of essential oil (EO) yield (content, % in biomass) and the concentration of various constituents (% of total oil) in hemp EO from this study and the literature reports.

	Wild Hemp	Reg. cvs	New Lines	[10]	[14]	[17]	[20]	[12]	[22]	[13]	[19]	[23]	[24]	[18]
Extraction type		HD	HD	HD	SD	HD	HD	SE	SD	HD	HS-SPME	SE	SD/HD	HD
Oil content/EO yields	0.09–0.26	0.1–0.2	0.06–0.14	0.11–0.25%	0.23–0.31%	0.1–0.3%	-	-	0.3%	0.28%	-	-	0.04–0.12%	-
α-Pinene	0–2.5	0–8.4	0.15–2.9	3–20%	10.9–16.99	2–7.8%		23%	17.9%	11%	9.6–40.1%		8.1–18.2%	1.6–2.9%
β-Pinene	0–0.98	0.06–2	0–1.4	1–8%	6.38–9.33			8.6%			2.9–9.3%		2.6–5.2%	
Isocaryophyllene (γ-Caryophyllene)	0.2–1.2	0.6–1.4	0.6–1.6											
β-Caryophyllene	15.4–29.7	22.4–55	11.4–22	7–28%	10.56–13.90		18.7%	46%			5.2–22.6%	4.83–5.8 mg mL^−1^		
α-(E)-Bergamotene	0.75 to 1.9	3.9–6.8	8.9–17			1.8–2.7		3.6%		4%		1.54–1.91 mg mL^−1^		1.9–2.7%
(Z)-β-Farnesene	0.13–3.0	0–2.8	0.4–2.1					4.4%				2.37–2.91 mg mL^−1^	1.4–3.0%	
Caryophyllene oxide	0.24–31.2	3.9–6.8	8.7–17	2–6%		3–11				15%			1.2–13%	2.3–4.5%
Humulene epoxide 2	1.3–3.2	0.4–2.3	2.3–5.6											
Selina-6-en-4-ol	0.23 to 1.6	0–1.8	1.1–2.8											
Caryophylla-4(12),8(13)-dien-5α-ol	1.1–5.4	0–2.3	2.3–6.6											0.7–2.0%
Caryophylla-4(12),8(13)-dien-5β-ol	1.1–5.4	2.3	2.3–6.6											
14-hydroxy-(Z)-Caryophyllene	0–3.4	0–1.3	0.72–2.1											
β-Bisabolol	0.9–4.5	0–1.8	2.9–4.0											
α-Bisabolol	0.4–.9	0–6.9	0.5–3.5											
Cannabidiol, CBD	6.9–52.4	7.1–25	6.4–25	0.9–4.4%		10.0–11.1					1.9–8.6mg/g	0.1–0.15%	0.1–7.6%	24.9%
THC, δ9-Tetrahydrocannabinol	0–3.4	0–1.18	0.4–3.6											
Monoterpenes	0–8.03	0.3–13	0.19–10		58.06–68.4	5.3	57.2%		54.2%		47–89%		26.7–54%	2–7%
Sesquiterpenes	40.9–88.3	65–89	70–89		26.11–37.97	75	40.4%		46%	67%			45.1–52.6%	65–75%
Cannabinoids	6.0–56.3	4.6–28	6.4–27			10.2			0.1%				0.1–7.9%	11–24%
E-ocimene/trans-Ocimene				1–10%				10%			0.4–7.1%	2.23–3.42 mg mL^−1^	2.1–5.1%	
α- and β Myrcene				8–45%	12.5–29.2	11.3	22.9%	27%	25%	11%	6.9–37%	1.8–3.2 mg mL^−1^	7.1–14.2%	
Terpinolene				0.12–22%	3.4–10.7		12%	18.7%		6%			7.8–10%	
α-Humulene				3–12%		7.1–8.9		19%		13%	1.3–8.7%	1.55–1.97 mg mL^−1^	5–11.2%	9%
(E)-Caryophyllene						21.4–26			45.4%	28%			14–32.7%	24.5–29%
α- and β-Selinene										7%				4.3–7.3%
Limonene					3.11–4.99			12%			1.6–11.5%			
Citral												0.09–3.5 mg mL^−1^		

Hydro-distillation (HD); Steam-distillation (SD); Headspace solid-phase microextraction (HS-SPME); Solvent extraction (SE); Microwave-assisted extraction (MAE).

**Table 4:** List of the industrial hemp cultivars and breeding lines used in this study.

Cultivar/Breeding Lines	Origin	Type	Registered	Plant Height, cm
CS	EU/Italy	dioecious	Yes	318
Spic	Serbia	monoecious	No	226
Dioica	EU/France	dioecious	Yes	298
Helena	Serbia	monoecious	Yes	302
Carmagnola	EU/Italy	dioecious	Yes	326
Sequieni	Romania	monoecious	Yes	278
Bacalmas	Hungary	dioecious	Landrace	312
Simba	Serbia	dioecious	No	256
Silesia	Poland	monoecious	Yes	268
Chameleon	Netherlands	dioecious	Yes	312
Fibrol	Hungary	monoecious	Yes	-
Futura	France	monoecious	Yes	295
Lovrin	Romania	dioecious	Yes	289

EU—European Union List.

**Table 5:** Wild hemp (*C. sativa* L.) sample collection locations.

Location	GPS
Buro	N5070877 E459221
Daleka Zemlia	N5019382 E395825
Paluka	N5083581 E344444
Perez	N4989836 E526695
Saykaj	N5011419 E429982
Susara	N4975406 E510258
Slavka	N4994987 E478790
Kovacica	N4954007 E472403
Titelski Breg	N5012250 E436909

## Supplementary Materials

The following are available online, Table S1. Essential oil constituents of wild hemp accessions, in % of total oil. Table S2. Essential oil constituents of new hemp breeding lines in % of total oil (area under the curve). Table S3. Essential oil constituents of registered hemp cultivars.
