# Supplementary material for: Industrial, CBD, and Wild Hemp: How Different Are Their Essential Oil Profile and Antimicrobial Activity?
Source: Molecules. 2020 Oct 12;25(20):4631. doi: 10.3390/molecules25204631 (PMC7587197; doi:10.3390/molecules25204631)
Supplement: Supplementary file 1 [file molecules-25-04631-s001.pdf]

Industrial, CBD, and wild hemp: how different are their essential oil profile and antimicrobial activity?

Valtcho D. Zheljazkov[1]*, Vladimir Sikora[2], Ivayla Dincheva[3], Miroslava Kačániová[4], Tess Astatkie[5], Ivanka Semerdjieva[6], Dragana Latkovic[7]

[1] Crop and Soil Science Department, 3050 SW Campus Way, Oregon State University, Corvallis, OR 97331, U.S.A. Valtcho.jeliazkov@oregonstate.edu

[2] Institute for Field and Vegetable Crops, Alternative Crops and Organic Production Department, Maksima Gorkog 30, 21000 Novi Sad, Serbia, vladimir.sikora@ifvcns.ns.ac.rs

[3] Plant Genetic Research Group, Agrobioinstitute, Agricultural Academy, 8 "Dragan Tsankov" Blvd. 1164 Sofia, Bulgaria, ivadincheva@yahoo.com

[4] Department of Fruit Science, Viticulture and Enology, Faculty of Horticulture and Landscape Engineering, Tr. A. Hlinku 2, Slovak University of Agriculture in Nitra, 949 76 Nitra, Slovak Republic

[4] Department of Bioenergetics and Food Analysis, Institution of Food Technology and Nutrition, University of Rzeszow, Cwiklinskiej 1, 35-601 Rzeszow, Poland; kacaniova.miroslava@gmail.com

[5] Department of Engineering, Faculty of Agriculture, Dalhousie University, Truro, NS, B2N 5E3, Canada astatkie@dal.ca

[6] Department of Botany and Agrometeorology, Faculty of Agronomy, Agricultural University, Plovdiv, Bulgaria, v_semerdjieva@abv.bg

[7] University of Novi Sad, Department of Field and Vegetable Crops, Dr Zorana Đinđića 1, 21000 Novi Sad, Serbia, dragana.latkovic@polj.uns.ac.rs

* Correspondence: ValtchoJeliazkov@oregonstate.edu; Tel.: 1-541-737-5877)

Supplementary Material

Supplemental Table 1. Essential oil constituents of wild hemp accessions, in % of total oil.

RI	Name	Class	811	812	813	814	815	816	817	818	819
			**Slavka**	**Kovacica**	**Buro**	**Daleka zemlia**	**Susara**	**Saykaj**	**Perez**	**Titelski breg**	**Paluka**
		**Oil content, volume**		**0.115**	**0.271**	**0.085**	**0.262**	**0.100**	**0.185**	**0.156**	**0.130**
932	**α-Pinene**	bicyclic monoterpene	2.448	0.118	nd	nd	2.316	nd	1.293	1.745	0.497
974	β-Pinene	bicyclic monoterpene	0.981	nd	nd	nd	0.928	nd	0.425	0.699	0.424
988	Myrcene	bicyclic monoterpene	nd	nd	nd	0.569	0.122	nd	0.630	0.294	1.032
1002	α-Phellandrene	monocyclic monoterpene	nd	nd	nd	nd	nd	nd	nd	nd	0.138
1007	δ-3-Carene	bicyclic monoterpene	nd	nd	nd	nd	nd	nd	nd	nd	0.617
1023	Limonene	monocyclic monoterpene	0.763	nd	nd	0.270	0.722	nd	0.154	1.244	0.128
1026	Eucalyptol	bicyclic monoterpenoid	0.572	nd	nd	nd	0.541	nd	1.546	0.302	0.209
1032	β-(Z)-Ocimene	acyclic monoterpene	0.101	nd	nd	nd	0.107	nd	0.146	0.094	0.174
1044	β-(E)-Ocimene	acyclic monoterpene	0.306	nd	nd	0.740	0.290	nd	0.507	0.150	1.633
1055	γ-Terpinene	monocyclic monoterpene	nd	nd	nd	nd	0.084	nd	0.286	0.029	0.161
1066	(Z)-Sabinene hydrate	bicyclic monoterpenoid	nd	nd	nd	nd	0.101	nd	0.328	0.053	nd
1087	Terpinolene	monocyclic monoterpene	nd	nd	nd	0.377	0.079	nd	0.343	0.068	3.017
1098	(E)-Sabinene hydrate	bicyclic monoterpenoid	nd	nd	nd	nd	0.082	nd	0.155	0.044	nd
1114	endo-Fenchol	bicyclic monoterpenoid	nd	nd	nd	nd	0.088	nd	nd	0.086	nd
1117	exo-Fenchol	bicyclic monoterpenoid	nd	nd	nd	nd	0.056	nd	nd	0.059	nd
1135	(E)-Pinocarveol	bicyclic monoterpenoid	nd	nd	nd	nd	0.049	nd	nd	0.027	nd
1166	Borneol	bicyclic monoterpenoid	nd	nd	nd	nd	0.045	nd	0.034	0.029	nd
1181	(Z)-Carveol	monocyclic monoterpenoid	nd	nd	nd	nd	0.026	nd	0.132	nd	nd
1188	α-Terpineol	monocyclic monoterpenoid	nd	nd	nd	nd	nd	nd	0.089	nd	nd
1290	(E)-Verbenyl acetate	bicyclic monoterpenoid	nd	nd	nd	nd	nd	nd	0.043	nd	nd
1388	β-Bourbonene	tricyclic sesquiterpene	nd	nd	nd	nd	0.048	nd	nd	nd	0.452
1393	7-epi-Sesquithujene	bicyclic sesquiterpene	nd	nd	nd	nd	0.035	nd	0.071	0.043	0.460
1407	**Isocaryophyllene (γ-**	bicyclic sesquiterpene	1.225	0.582	0.288	0.358	1.159	0.725	0.627	0.953	0.200
1411	α-(Z)-Bergamotene	bicyclic sesquiterpene	0.274	0.213	0.411	0.176	0.259	0.171	0.113	0.336	0.283
1418	**β-Caryophyllene**	bicyclic sesquiterpene	24.348	29.688	16.329	15.404	26.824	18.588	22.404	25.918	24.518
1430	**α-(E)-Bergamotene**	bicyclic sesquiterpene	1.615	0.753	1.255	0.749	1.528	1.130	1.003	1.888	1.756
1435	β-Gurjunene	bicyclic sesquiterpene	0.291	0.182	0.288	nd	0.275	0.179	0.234	0.243	0.204
1441	**(Z)-β-Farnesene**	acyclic sesquiterpene	1.859	0.405	0.127	0.461	1.759	1.186	1.015	2.258	3.011
1452	α-Humulene (α-	monocyclic sesquiterpene	8.646	10.541	5.694	5.361	11.966	6.522	9.544	10.909	11.020
1457	L-alloaromadendrene	tricyclic sesquiterpene	0.823	1.210	0.556	1.200	0.779	1.103	1.880	0.758	1.293
1470	4,5-di-epi-Aristolochene	bicyclic sesquiterpene	0.203	nd	nd	nd	0.192	0.228	0.248	0.217	0.088
1474	(E)-Cadina-1(6),4-diene	bicyclic sesquiterpene	0.280	nd	nd	nd	0.265	0.219	0.373	0.296	0.143
1480	γ-Himachalene	bicyclic sesquiterpene	0.346	0.192	0.101	nd	0.327	0.252	0.362	0.429	0.160
1489	β-Selinene	bicyclic sesquiterpene	1.404	0.437	0.371	0.139	1.329	0.855	2.217	1.587	0.951
1492	δ-Selinene	bicyclic sesquiterpene	3.651	3.048	1.374	0.533	3.455	4.768	5.329	4.187	2.579
1497	α-Selinene	bicyclic sesquiterpene	0.628	0.093	0.176	nd	0.594	0.103	1.022	0.641	0.376
1501	β-Himachalene	bicyclic sesquiterpene	2.122	2.185	0.928	0.430	2.008	3.092	3.164	2.622	1.756
1513	γ-Cadinene	bicyclic sesquiterpene	0.753	0.128	nd	nd	0.713	0.147	0.849	0.675	1.320
1522	δ-Cadinene	bicyclic sesquiterpene	1.037	0.176	0.254	0.150	0.981	0.362	0.257	0.788	0.701
1536	α-Cadinene	bicyclic sesquiterpene	0.379	0.097	nd	0.276	0.359	0.232	0.235	0.232	0.174
1544	Selina-3,7(11)-diene	bicyclic sesquiterpene	0.116	0.266	0.187	nd	0.110	0.196	0.476	0.456	0.460
1555	(E)-Cadinene ether	bicyclic sesquiterpenoid	0.000	nd	nd	nd	nd	nd	nd	0.208	0.536
1561	Nerolidol	acyclic sesquiterpenoid	0.302	0.312	0.106	nd	0.286	0.335	0.202	0.424	1.297
1566	Maaliol	bicyclic sesquiterpenoid	0.416	1.396	nd	0.264	0.394	0.097	0.188	0.316	0.516
1570	n-Tridecanol	fatty alcohol	0.788	nd	nd	0.133	0.746	0.360	1.157	0.923	0.233
1572	Germacrene D-4-ol	monocyclic sesquiterpenoid	0.206	1.940	0.213	0.491	0.195	0.102	0.223	0.538	0.424
1576	Spathulenol	tricyclic sesquiterpenoid	0.295	0.227	0.386	0.700	0.279	0.197	0.154	0.172	2.531
1578	Himachalene epoxide	tricyclic sesquiterpenoid	0.146	0.166	nd	0.243	0.138	0.048	0.106	0.181	0.638
1581	Hexyl octanoate	ester of fatty acid	0.205	nd	nd	0.558	0.194	0.122	0.144	0.174	0.236
1584	**Caryophyllene oxide**	bicyclic sesquiterpenoid	6.606	4.844	5.215	31.200	6.251	6.103	4.608	5.193	0.240
1590	Globulol	bicyclic sesquiterpenoid	0.224	0.187	0.138	0.955	0.212	0.177	1.006	0.150	7.672

1601	Ledol	tricyclic sesquiterpenoid	0.361	0.224	0.174	1.322	0.342	0.259	0.204	0.267	0.206
1606	Dodecyl acetate	ester	0.467106	0.134	0.253	0.862	0.442	0.463	0.608	0.351	0.787
**1608**	**Humulene epoxide 2**	**bicyclic sesquiterpenoid**	**2.078**	**1.507**	**1.595**	**9.517**	**1.966**	**1.699**	**1.299**	**1.637**	**3.162**
1615	β-Himachalene oxide	tricyclic sesquiterpenoid	0.502	0.225	0.177	0.353	0.475	0.315	0.355	0.396	0.358
**1626**	**Selina-6-en-4-ol**	**bicyclic sesquiterpenoid**	**0.479**	**0.343**	**0.228**	**0.915**	**0.453**	**0.280**	**0.577**	**0.516**	**1.618**
1637	**Caryophylla-**	bicyclic sesquiterpenoid	2.306	1.707	0.975	3.258	2.182	1.747	1.346	1.638	1.310
1639	**Caryophylla-**	bicyclic sesquiterpenoid	2.728	2.558	1.521	5.383	2.581	2.540	1.589	1.864	1.128
1663	14-hydroxy-(Z)-	bicyclic sesquiterpenoid	0.109	0.154	nd	3.372	0.103	nd	0.181	0.203	0.237
1668	14-hydroxy-9-epi-(E)-	bicyclic sesquiterpenoid	1.694	1.807	0.797	nd	1.603	1.460	1.764	1.261	0.697
1670	epi-β-Bisabolol	monocyclic sesquiterpenoid	0.481	nd	nd	nd	0.455	0.538	1.742	0.550	0.409
1675	**β-Bisabolol**	monocyclic sesquiterpenoid	1.989	1.705	0.898	4.506	1.882	1.991	1.349	1.555	1.766
1686	α-Bisabolol	monocyclic sesquiterpenoid	2.858	0.435	0.134	0.585	2.704	0.690	1.157	1.218	1.059
1694	Eudesm-7(11)-en-4-ol	bicyclic sesquiterpenoid	0.119	0.236	nd	nd	0.113	0.073	0.451	0.268	1.119
1822	(E)-Nerolidyl	acyclic sesquiterpenoid	0.096	nd	nd	nd	0.091	0.069	0.075	0.143	0.093
1943	Phytol	acyclic diterpenoid	0.000	nd	nd	nd	nd	nd	nd	nd	0.170
2095	Methyl linoleate	ester of unsaturated fatty	0.398	0.699	0.510	nd	0.377	0.250	0.903	1.049	nd
2132	Linoleic acid	unsaturated fatty acid	0.166	nd	nd	nd	0.157	0.100	0.104	0.195	0.101
2283	ẟ9-	cannabinoid	0.167	0.394	nd	nd	0.158	nd	0.105	0.085	nd
2306	**Cannabicyclol**	cannabinoid	0.117	0.088	0.282	nd	0.111	0.383	0.088	0.097	0.095
2381	**CBD**	cannabinoid	14.880	22.649	52.421	6.931	11.295	33.685	17.523	15.760	7.872
2399	Cannabichromene	cannabinoid	0.507	0.670	1.881	nd	0.480	1.572	0.563	0.502	0.157
2462	ẟ8-	cannabinoid	0.084	nd	0.342	nd	0.131	0.265	0.163	0.106	0.191
2484	**ẟ9-**	cannabinoid	1.564	3.397	1.417	nd	1.480	2.580	0.592	0.445	1.899
		**Monoterpenes**	5.170	0.118	0.000	1.956	5.636	0.000	6.111	4.923	8.030
		**Sesquiterpenes**	73.990	70.169	40.896	88.301	77.670	58.778	70.071	71.134	78.993
		**cannabinoid**	17.319	27.198	56.343	6.931	13.655	38.485	19.034	16.995	10.214
		**Other(acyclic diterpenoids;**	2.024	0.833	0.753	1.553	1.916	1.295	2.916	2.692	1.527

Supplemental Table 2. Essential oil constituents of new hemp breeding lines in % of total oil (area under the curve)

RI	Name	903	904	905	906	907	908	909	910
		SK 1, 2019	SK 2, 2019	SK 3, 2019	SK 4, 2019	SK 5, 2019	SK 6, 2019	SK 7, 2019	SK 8, 2019
		**0.06**	**0.12**	**0.1**	**0.12**	**0.1**	**0.14**	**0.1**	**0.14**
932	**α-Pinene**	0.219	2.950	0.155	0.434	1.089	1.422	0.188	0.908
974	β-Pinene	0.138	1.370	nd	0.194	0.447	0.569	Nd	0.551
988	Myrcene	nd	0.664	nd	0.233	0.526	0.384	Nd	0.275
1002	α-Phellandrene	nd	0.527	nd	nd	nd	nd	Nd	nd
1007	δ-3-Carene	nd	nd	nd	nd	nd	nd	Nd	nd
1023	Limonene	0.214	1.051	nd	0.092	0.145	0.939	Nd	0.425
1026	Eucalyptol	0.233	1.096	0.198	0.127	0.261	0.284	Nd	0.478
1032	β-(Z)-Ocimene	nd	0.206	nd	nd	nd	nd	Nd	nd
1044	β-(E)-Ocimene	nd	0.990	nd	0.232	0.374	0.209	Nd	0.074
1055	γ-Terpinene	nd	0.174	nd	nd	nd	nd	Nd	0.069
1066	(Z)-Sabinene hydrate	nd	0.223	0.125	nd	nd	nd	Nd	0.130
1087	Terpinolene	nd	0.299	nd	0.336	0.190	0.543	Nd	0.243
1098	(E)-Sabinene hydrate	nd	0.182	0.132	nd	nd	nd	Nd	0.119
1114	endo-Fenchol	nd	0.118	nd	nd	nd	0.177	Nd	0.096
1117	exo-Fenchol	nd	0.083	nd	nd	nd	0.100	Nd	0.060
1135	(E)-Pinocarveol	nd	0.101	nd	0.219	0.191	nd	Nd	0.045
1166	Borneol	nd	0.108	nd	0.060	nd	0.097	Nd	0.057
1181	(Z)-Carveol	nd	0.148	0.130	0.134	nd	nd	Nd	0.073
1188	α-Terpineol	nd	0.111	nd	0.081	nd	nd	Nd	0.047
1290	(E)-Verbenyl acetate	nd	nd	nd	0.064	nd	nd	Nd	0.042
1388	β-Bourbonene	nd	nd	nd	0.049	nd	nd	Nd	0.091
1393	7-epi-Sesquithujene	nd	nd	nd	nd	nd	nd	Nd	0.174
1407	**Isocaryophyllene (γ-**	**1.629**	**0.962**	**0.973**	**0.575**	**0.869**	**1.003**	**0.783**	**0.802**
1411	α-(Z)-Bergamotene	0.477	0.078	0.139	0.294	0.337	0.465	0.180	0.333
1418	**β-Caryophyllene**	**21.561**	**16.391**	**15.093**	**13.421**	**14.617**	**16.753**	**16.281**	**11.380**
1430	**α-(E)-Bergamotene**	**1.959**	**0.563**	**1.137**	**1.776**	**1.903**	**2.754**	**1.137**	**1.081**
1435	β-Gurjunene	0.306	0.214	0.193	0.327	0.401	0.361	0.202	1.384
1441	(Z)-β-Farnesene	1.914	0.492	0.896	1.535	1.624	2.545	1.188	2.097
1452	α-Humulene (α-Caryophyllene)	7.638	6.682	6.028	5.799	6.411	6.855	5.484	4.438
1457	L-alloaromadendrene	1.173	0.547	0.616	2.211	1.146	0.990	0.750	0.706
1470	4,5-di-epi-Aristolochene	nd	0.204	0.101	0.120	nd	nd	Nd	0.279
1474	(E)-Cadina-1(6),4-diene	nd	0.282	0.138	0.648	0.257	0.115	Nd	0.715
1480	γ-Himachalene	0.699	0.451	0.506	0.346	0.382	0.337	Nd	0.316
1485	Germacrene D	0.300	0.153	0.150	0.137	nd	0.330	Nd	0.227
1489	β-Selinene	0.472	1.279	0.317	0.422	0.574	0.852	0.317	0.805
1492	δ-Selinene	1.317	4.044	1.619	2.367	1.976	2.201	0.951	2.328
1497	α-Selinene	0.714	0.659	0.253	0.169	0.231	0.482	0.281	0.398
1501	β-Himachalene	0.633	2.446	1.070	1.471	1.038	1.493	0.567	1.658
1513	γ-Cadinene	0.267	0.788	0.572	0.238	0.279	0.554	0.297	0.571
1522	δ-Cadinene	0.708	0.276	0.750	0.483	0.449	0.686	0.410	1.066
1536	α-Cadinene	0.585	0.420	0.209	0.700	0.373	0.592	0.278	1.352
1544	Selina-3,7(11)-diene	0.217	0.557	0.334	0.685	0.288	0.500	0.671	0.597
1555	(E)-Cadinene ether	0.813	0.290	0.450	0.946	1.217	0.829	0.568	0.909
1561	Nerolidol	0.277	0.569	0.351	0.223	0.436	1.536	0.627	1.587
1570	Caryophyllenyl alcohol	0.258	1.192	0.941	0.178	0.808	0.819	0.259	0.804
1571	Caryolan-8-ol	0.193	0.967	1.210	0.372	0.764	0.460	0.823	2.000
1573	Germacrene D-4-ol	0.267	0.107	1.260	0.205	0.465	1.090	0.661	0.228
1576	Spathulenol	1.122	0.850	0.548	0.727	0.911	0.639	0.752	1.650
1578	Himachalene epoxide	0.562	0.299	0.218	0.504	0.472	0.838	0.674	0.731
1581	Hexyl octanoate	0.621	0.417	0.226	0.744	0.643	0.727	0.668	0.507

1584	**Caryophyllene oxide**	**13.795**	**10.950**	**9.569**	**13.461**	**16.650**	**11.670**	**12.246**	**8.682**
1590	Globulol	0.840	0.594	0.472	0.609	0.785	0.679	0.622	1.493
1601	Ledol	0.771	0.624	0.546	0.636	0.776	0.557	0.531	0.456
1606	Dodecyl acetate	1.277	0.513	0.685	1.875	1.744	1.086	0.952	0.883
1608	**Humulene epoxide 2**	**4.785**	**5.178**	**3.480**	**4.654**	**5.641**	**3.537**	**3.830**	**2.338**
1615	β-Himachalene oxide	0.873	0.955	0.688	0.550	1.000	0.989	0.682	1.362
1626	**Selina-6-en-4-ol**	**1.508**	**1.771**	**1.859**	**1.187**	**2.152**	**2.775**	**1.854**	**2.791**
1637	**Caryophylla-4(12),8(13)-dien-5α-ol**	4.884	4.160	3.540	3.370	4.300	3.138	3.076	2.400
1639	**Caryophylla-4(12),8(13)-dien-5β-ol**	6.202	5.935	3.466	4.744	5.560	3.222	4.243	2.371
1663	14-hydroxy-(Z)-Caryophyllene	0.773	1.043	1.095	0.647	0.744	1.417	0.719	2.078
1668	**14-hydroxy-9-epi-(E)-**	3.605	0.808	3.676	2.692	3.766	2.749	3.195	4.496
1670	epi-β-Bisabolol	0.295	0.762	0.618	0.403	0.493	0.403	0.294	1.579
1675	**β-Bisabolol**	**4.031**	**3.242**	**3.193**	**3.634**	**3.892**	**2.987**	**3.048**	**3.142**
1686	**α-Bisabolol**	**0.542**	**0.968**	**3.886**	**1.099**	**0.609**	**1.024**	**0.753**	**3.484**
1694	Eudesm-7(11)-en-4-ol	0.168	0.542	0.161	0.087	0.286	1.097	0.550	1.392
1822	(E)-Nerolidyl isobutyrate	Nd	0.183	0.359	0.123	nd	0.142	Nd	0.225
1943	Phytol	Nd	nd	0.544	0.245	nd	nd	Nd	0.317
2095	Methyl linoleate	Nd	0.113	0.509	1.156	0.157	0.138	0.545	0.759
2132	Linoleic acid	Nd	nd	nd	nd	nd	nd	Nd	0.218
2283	**δ9-Tetrahydrocannabivarin**	Nd	0.102	0.168	0.224	nd	nd	Nd	1.200
2306	**Cannabicyclol**	Nd	nd	0.219	0.285	nd	nd	0.243	0.491
2381	**CBD**	6.405	6.956	21.533	16.051	7.676	8.954	25.392	6.832
2399	**Cannabichromene**	Nd	0.179	0.681	0.561	nd	0.201	0.587	0.724
2462	**δ8-Tetrahydrocannabinol**	Nd	nd	0.246	0.083	nd	nd	Nd	0.173
2484	**δ9-Tetrahydrocannabinol**	Nd	0.274	0.384	0.256	nd	nd	0.286	3.624
	**Monoterpenes**	0.804	10.401	0.740	2.206	3.223	4.724	0.188	3.692
	**Sesquiterpenes**	89.133	79.477	72.680	74.824	84.882	82.464	69.784	78.996
	**cannabinoid**	6.405	7.511	23.231	17.460	7.676	9.155	26.508	13.044
	**Other (acyclic diterpenoids; ester;**	1.898	1.043	1.964	4.020	2.544	1.951	2.165	2.684

Supplemental Table 3. Essential oil constituents of registered hemp cultivars.

RI		**CS**	**ŠPIC**	**Dioica**	**Helena**	**Carmagnola**	**Sequieni**	**Bacalmas**	**Simba**
		0.10	0.10	0.17	0.11	0.11	0.14	0.12	0.13
924	α-Thujene	nd	nd	nd	nd	Nd	nd	nd	nd
**932**	**α-Pinene**	nd	5.15	0.36	2.41	1.53	2.51	3.21	2.80
946	Camphene	nd	nd	nd	nd	Nd	nd	nd	nd
969	Sabinene	nd	nd	nd	nd	Nd	nd	nd	nd
**974**	**β-Pinene**	nd	2.21	0.20	1.12	0.76	0.97	1.40	1.18
988	Myrcene	nd	nd	nd	1.75	0.51	1.38	0.31	1.19
1002	α-Phellandrene	nd	nd	nd	nd	Nd	nd	nd	nd
1007	δ-3-Carene	nd	nd	0.79	nd	0.19	nd	nd	0.45
1014	α-Terpinene	nd	nd	nd	nd	Nd	nd	nd	nd
1020	p-Cymene	nd	nd	nd	nd	Nd	nd	nd	nd
1023	Limonene	nd	nd	nd	0.92	0.59	1.23	0.29	1.29
1026	Eucalyptol	nd	2.30	nd	1.12	0.73	0.93	2.38	1.30
1032	β-(Z)-Ocimene	nd	nd	nd	nd	Nd	nd	nd	nd
1044	β-(E)-Ocimene	nd	nd	nd	nd	Nd	nd	nd	nd
1055	γ-Terpinene	nd	nd	nd	nd	Nd	nd	0.33	0.15
1066	(Z)-Sabinene hydrate	nd	nd	nd	0.19	Nd	nd	0.55	0.28
1087	Terpinolene	nd	nd	nd	nd	Nd	nd	nd	nd
1098	(E)-Sabinene hydrate	nd	nd	nd	nd	Nd	nd	0.31	0.26
1114	endo-Fenchol	nd	nd	nd	nd	nd	nd	nd	0.14
1117	exo-Fenchol	nd	nd	nd	nd	nd	nd	nd	nd
1135	(E)-Pinocarveol	nd	nd	nd	nd	nd	nd	nd	nd
1140	(Z)-Pinene hydrate	nd	nd	nd	nd	nd	nd	nd	nd
1166	Borneol	nd	nd	nd	nd	nd	nd	nd	nd
1181	(Z)-Carveol	nd	nd	nd	nd	nd	nd	nd	nd
1184	3-Decanone	nd	nd	nd	nd	nd	nd	nd	nd
1188	α-Terpineol	nd	nd	nd	nd	nd	nd	nd	nd
1290	(E)-Verbenyl acetate	nd	nd	nd	nd	nd	nd	nd	nd
1346	2,6-Dimethoxyphenol	nd	nd	nd	nd	nd	nd	nd	nd
1360	Neryl acetate	nd	nd	nd	nd	nd	nd	nd	nd
1372	α-Ylangene	nd	nd	0.16	nd	nd	nd	nd	nd
1376	α-Copaene	nd	nd	nd	nd	nd	nd	nd	nd
1388	β-Bourbonene	nd	nd	nd	nd	0.09	nd	0.65	0.13
1393	7-epi-Sesquithujene	nd	nd	nd	nd	nd	nd	nd	nd
**1407**	**Isocaryophyllene (γ-**	0.96	nd	1.13	0.98	1.01	1.35	1.05	0.93
1411	α-(Z)-Bergamotene	nd	nd	0.20	0.54	0.27	0.24	0.45	0.26
**1418**	**β-Caryophyllene**	32.49	39.90	25.53	27.94	26.70	33.30	26.94	25.32
**1430**	**α-(E)-Bergamotene**	0.37	2.98	1.55	3.79	2.10	1.21	3.10	1.73
1435	β-Gurjunene	0.34	nd	0.38	0.29	0.40	0.36	0.24	0.35
**1441**	**(Z)-β-Farnesene**	0.32	nd	1.25	3.33	1.63	1.22	2.67	2.08
1452	α-Humulene (α-Caryophyllene)	17.42	14.11	11.40	11.49	12.15	14.29	10.51	12.53
1457	L-alloaromadendrene	1.18	1.87	1.85	1.42	1.57	2.72	1.22	1.39
1480	γ-Himachalene	nd	nd	0.22	0.28	0.39	nd	0.30	0.38
1489	β-Selinene	nd	nd	0.51	0.61	0.68	0.45	1.61	0.62
1492	δ-Selinene	0.80	1.38	1.31	1.66	2.14	2.02	2.89	2.20
1497	α-Selinene	nd	0.81	0.32	0.27	0.30	0.70	0.93	0.31
1501	β-Himachalene	0.48	nd	0.88	1.24	1.63	0.79	1.45	1.61
1513	γ-Cadinene	0.40	nd	0.30	0.41	0.47	0.45	0.29	0.52
1522	δ-Cadinene	1.01	nd	0.52	0.42	1.20	nd	0.76	1.11
1536	α-Cadinene	nd	nd	0.41	0.35	0.45	0.34	0.49	0.45
1544	Selina-3,7(11)-diene	nd	nd	0.39	0.44	0.42	0.32	0.37	0.43
1561	Nerolidol	0.33	nd	1.07	0.59	0.66	0.28	0.52	0.51
1566	Maaliol	nd	nd	0.35	0.68	1.70	nd	0.20	1.18
1570	n-Tridecanol	1.31	nd	1.87	1.58	1.19	0.18	1.00	1.15

1572	Germacrene D-4-ol	0.29	nd	1.30	0.78	1.95	nd	0.24	1.64
1576	Spathulenol	nd	nd	0.51	0.27	0.49	nd	0.53	0.64
1578	Himachalene epoxide	nd	nd	0.55	0.33	0.19	nd	0.40	0.30
**1584**	**Caryophyllene oxide**	5.39	6.04	6.15	6.29	4.20	6.61	6.58	4.73
1590	Globulol	nd	nd	0.36	0.25	0.87	nd	0.43	0.35
1601	Ledol	0.32	nd	0.44	0.26	0.54	0.57	0.28	0.25
**1608**	**Humulene epoxide 2**	1.35	0.96	1.53	1.80	1.40	1.95	2.00	1.61
1615	β-Himachalene oxide	nd	nd	0.59	0.50	0.36	0.35	0.42	0.40
**1626**	**Selina-6-en-4-ol**	0.56	nd	1.59	0.89	1.31	nd	0.39	0.66
**1637**	**Caryophylla-4(12),8(13)-dien-**	1.15	nd	1.68	1.20	1.36	1.93	1.62	1.28
**1639**	**Caryophylla-4(12),8(13)-dien-**	1.14	nd	1.54	1.44	1.09	2.29	1.82	1.32
**1663**	**14-hydroxy-(Z)-Caryophyllene**	nd	nd	0.36	0.91	0.44	nd	0.37	0.42
1668	14-hydroxy-9-epi-(E)-	0.97	nd	1.66	1.11	2.31	1.41	1.13	1.52
**1675**	**β-Bisabolol**	1.06	nd	1.26	1.39	1.14	1.69	1.72	1.15
1686	α-Bisabolol	6.69	nd	2.10	1.75	4.06	0.50	2.66	4.15
1694	Eudesm-7(11)-en-4-ol	nd	nd	0.69	0.44	0.38	0.67	0.72	0.35
1822	(E)-Nerolidyl isobutyrate	1.42	nd	0.82	nd	1.10	nd	nd	0.54
1943	Phytol	nd	nd	nd	nd	nd	nd	nd	nd
2095	Methyl linoleate	nd	nd	0.75	0.38	1.02	0.36	nd	0.46
2132	Linoleic acid	0.32	nd	0.36	0.24	0.36	0.28	nd	nd
2283	δ9-Tetrahydrocannabivarin	nd	nd	nd	nd	0.52	nd	nd	nd
2306	Cannabicyclol	nd	nd	nd	nd	nd	nd	nd	nd
**2381**	**CBD**	16.05	7.94	19.32	10.64	9.85	10.66	9.75	10.36
2399	Cannabichromene	nd	nd	0.80	nd	0.36	nd	0.21	0.43
2462	δ8-Tetrahydrocannabinol	nd	nd	nd	nd	nd	nd	nd	nd
**2484**	**δ9-Tetrahydrocannabinol**	nd	nd	nd	nd	0.93	0.42	nd	0.76
	Monoterpenes	1.56	13.54	1.38	7.68	4.49	7.10	8.86	9.11
	**Sesquiterpenes**	78.05	77.27	72.97	76.79	79.21	79.23	78.12	75.46
	**cannabinoid**	16.54	7.94	20.68	11.05	11.36	11.24	9.96	11.72
	Other(acyclic diterpenoids;	2.40	0.61	3.23	2.40	2.77	0.82	1.40	1.95
